# Development of a Mixed-Strain Fermentation Process for Sour Pomegranate: An Analysis of Metabolites and Flavor Compounds

**DOI:** 10.3390/foods14213733

**Published:** 2025-10-30

**Authors:** Yuting Yang, Ailikemu Mulati, Xinmeng Huang, Yuanpeng Li, Dilireba Shataer, Haipeng Liu, Jiayi Wang

**Affiliations:** 1 Xinjiang Key Laboratory of Biological Resources and Genetic Engineering, College of Smart Agriculture (Research Institute), Xinjiang University, Urumqi 830046, China; 2 Centre for Intelligent Healthcare, Coventry University, Coventry CV1 5RW, UK

**Keywords:** sour pomegranate, mixed-strain fermentation, flavor compounds, metabolites

## Abstract

Sour pomegranate, a distinctive product of Xinjiang, China, is characterized by its sour and astringent taste, which contributes to a low processing rate. This study utilized single-factor experiments to screen three strains: *Lactobacillus fermentum*, *Lactobacillus plantarum*, and *Lactobacillus acidophilus*. Through uniform design experiments, the mixed-strain ratio of *L. fermentum*:*L. plantarum*:*L. acidophilus* = 45%:31%:28% was determined. In addition, the amount of mixed inoculum was 2%, and the fermentation time was 11 h. Additionally, a mixed inoculation amount of 2% and a fermentation duration of 11 h were established. Utilizing electronic nose, electronic tongue, gas chromatography–ion mobility spectrometry, and non-targeted metabolomics, comparative analyses were conducted on the flavors and metabolites pre- and post-fermentation. The findings indicated that post-fermentation, umami increased by 32%, richness was enhanced by 6%, and the positive aftertaste was significantly extended. Mixed-strain fermentation facilitated the enrichment of alkanes, alcohols, aldehydes, and terpene volatile compounds; notably, the content of hexanal (fresh fruity aroma) and limonene (citrus aroma) increased by 1.95 times and 1.45 times, respectively, thereby augmenting the complexity of the aroma. Furthermore, mixed-strain fermentation significantly upregulated terpenes, amino acids and their derivatives, steroids and their derivatives, and alkaloid metabolites. These results offer potential technical support for the high-value utilization of agricultural products.

## 1. Introduction

Sour pomegranates, an important economic crop in Xinjiang, China [1,2], benefit from the region’s unique arid climate and significant temperature difference between day and night, providing ideal natural conditions for their growth and making them a signature agricultural product of the area. This fruit is rich in nutrients, including high levels of vitamin C, protein, 17 essential amino acids, and a variety of minerals [3]. The polyphenolic compounds in sour pomegranates include anthocyanins, proanthocyanidins, phenolic acids, flavonoid glycosides, and hydrolyzable tannins [4]. Nevertheless, due to its sour and astringent taste, its rate of processing and utilization has remained low for a long time. Most sour pomegranates are used only as feed, fertilizer, or even discarded as waste, which not only limits the increase in their added value but also causes pollution to the environment [5].

Fermentation is a method used to enhance the value of agricultural products and alter their flavor and nutritional properties. Numerous studies have been conducted on pomegranate fermentation. For example, studies focusing on pomegranate wine have explored the evolution of its antioxidant properties and flavor characteristics during fermentation [6]. In terms of antioxidant properties, the total phenolic content and free radical scavenging activity exhibited slight fluctuations during fermentation but maintained relatively high and stable levels during aging. Punicalagin and gallic acid, the main phenolic components, play a dominant role throughout the fermentation process. Regarding flavor characteristics, the most notable changes occurred in the early stages of fermentation (0–4 d), during which the aldehyde and ketone content decreased, while the ester and alcohol content increased. In addition, researchers have found that when pomegranate beverages are fermented with yeast, the total soluble solids (TSS) content decreases after fermentation, the ethanol content increases with the rise in initial TSS, and the titratable acidity increases [7]. However, to the best of our knowledge, there have been no reports on research related to the fermentation of sour pomegranates.

Lactic acid bacteria play crucial roles in the food industry and human health. Their application in the fermentation of fruit and vegetable juices not only imparts unique flavors to the products but also enhances their functionality [8]. However, fermentation with a single strain has certain limitations, such as low efficiency in utilizing reducing sugars and bioactive components, such as total phenols and flavonoids. In contrast, mixed-strain fermentation, which leverages the synergistic effects of multiple strains, has advantages over single-strain fermentation products in terms of physico-chemical properties, bioactivity, flavor, and nutritional value, and is therefore regarded as an ideal fermentation strategy [9]. For example, orange juice fermented with mixed-strain can significantly improve the retention rate of vitamin C and increase the content of flavor compounds, such as esters and alcohols, thereby enhancing the taste of the product [10]. Apple juice that has undergone mixed-strain fermentation exhibits significant improvements in both antioxidant activity and flavor [11].

This study used pomegranate juice produced in Xinjiang as the raw material and advanced the ratio of mixed strains composed of *L. fermentum*, *L. plantarum*, and *L. acidophilus*. In addition, electronic nose technology and gas chromatography–ion mobility spectrometry (GC-IMS) were used to analyze the aroma components after fermentation, and electronic tongue technology was used to evaluate changes in taste. In addition, changes in metabolite levels were analyzed using a non-targeted metabolomics approach. The results of this study are expected to provide a scientific basis for the high-value utilization of sour pomegranate resources, thereby promoting sustainable development of the food industry.

## 2. Materials and Methods

### 2.1. Materials and Strains

Sour pomegranate juice was provided by Jianongyunlian Co. Ltd. (Atushi, China), and stored in a −80 °C freezer for future use. The strains used in this study were *L. fermentum* CICC 25124, *L. casei* CICC 6114, *L. rhamnosus* CICC 6164, *L. acidophilus* CICC 6086, and *L. plantarum* CICC 25125, all obtained from the China Center of Industrial Culture Collection. The strains were preserved in 20% glycerol at −80 °C.

### 2.2. Optimization of Mixed-Strain Fermentation Conditions

Experiments were conducted to screen the strains, fermentation times, and inoculation ratios. Pomegranate juice was placed in water at 85 °C for 30 min to inactivate enzymes and microorganisms. Five types of lactic acid bacteria were cultured in De Man, Rogosa and Sharpe liquid medium at an inoculation rate of 1% (*v*/*v*), with each incubation carried out at 37 °C for 24 h in a constant temperature incubator, ensuring that the viable cell count of the bacterial suspension reached 1 × 10^7^ CFU/mL [12]. Based on different inoculation amounts (1%, 2%, 3%, 4%, and 5%), the five activated lactic acid bacteria were inoculated into the pomegranate juice and incubated on a shaker at 37 °C (shaking speed 200 rpm) for various durations (6 h, 10 h, 12 h, 14 h, and 16 h). Three suitable strains were selected based on comparisons of their polyphenol, total acid, and soluble solid contents.

Subsequently, optimization experiments for the mixed-strain ratios were conducted, with *L. fermentum*, *L. plantarum*, and *L. acidophilus* chosen as mixed strains. A uniform experimental design with three factors and eight levels was used. Table S1 shows the effects of different inoculum sizes (1% *v*/*v*, 2% *v*/*v*, 3% *v*/*v*, 4% *v*/*v*, and 5% *v*/*v*) and fermentation durations (10 h, 11 h, 12 h, 13 h, and 14 h) on polyphenol, total acidity, and soluble solids contents were analyzed to determine the optimal mixed-strain ratio.

### 2.3. Determination of Soluble Solids, Total Acidity, and Polyphenols

The content of soluble solids was measured using a handheld refractometer (PAL-BXIACID F5; ATAGO CO., LTD., Tokyo, Japan), with the refractometer calibrated to zero using distilled water before testing, and the results expressed as °Brix. The total acidity was determined by acid-base titration using an indicator [13]. The Folin-phenol method was used to measure the polyphenol content [14]. Briefly, the sample was diluted to 5 mL with 80% methanol, and 50 μL of the supernatant was mixed with 3 mL distilled water and 250 μL Folin reagent. After a 6 min reaction, 50 μL of 20% sodium carbonate solution was added, and the mixture was incubated at room temperature in the dark for 90 min. The absorbance was measured at 765 nm. The results were expressed as mg gallic acid equivalents per 100 g of fruit juice.

### 2.4. Determination of Volatile Aroma Components

#### 2.4.1. Electronic Nose Analysis

The samples were measured using an electronic nose (C-PEN3; Ainowiteng Technology Development Co., Ltd., Beijing, China). Three independent biological replicates were tested for the pomegranate juice both before and after fermentation. For each replicate, 10 mL of pomegranate juice was placed in a 50 mL headspace vial, sealed, and equilibrated at 45 °C for 20 min to stabilize the headspace gas (DZKW-S-4; Beijing Yongguangming Medical Instrument Co., Ltd., Beijing, China). The detection parameter was set as follows: flow rate of 150 mL/min, measurement time of 90 s to obtain a stable response, and flushing time of 60 s to eliminate interference between samples. The sample preparation time was 5 s, and automatic zeroing time were 5 s [15].

#### 2.4.2. GC-IMS

A GC-IMS instrument (FlavorSpec 1H1-00053 GC-IMS: Gesellschaft fur Analytische Sensorsysteme mbH, Dortmund, Germany) was used. Three biological replicates were analysed for both pre- and post-fermentation pomegranate juice. Three milliliters of pomegranate juice, both prior to and following fermentation, was placed in 20 mL headspace glass sampling vials. After equilibration at 40 °C for 15 min, a heated autosampler syringe at 85 °C was used to inject 500 μL of headspace gas. The chromatographic column employed was WAX (length: 15 m, inner diameter: 0.53 mm), with the column temperature maintained at 60 °C; both the carrier gas and drift gas were nitrogen (N2), and the ion mobility spectrometer temperature was set at 45 °C. The automatic headspace sampler injection volume was 500 μL, the sample incubation time was 20 min (60 °C), the injection needle temperature was set to 85 °C, and the stirring speed during incubation was 500 rpm [16].

In this study, volatile compounds separated by GC-IMS were identified using the built in GC-IMS Library Search software (Version 2.2). The retention indices (RIs) of the separated components were calculated using external standards of ketones (C_4_–C_9_). Based on both retention and drift times, a two-dimensional comparison was performed against the NIST retention index database and the IMS drift time database integrated into the software. Compounds whose similarity values met the required threshold were considered potential candidates. Identification was further verified by confirming that the monomer and dimer ions of each compound shared the same CAS number and molecular formula. The reactant ion peak (RIP) served as a reference to eliminate interfering signals. Compounds without a database match were labeled as “unidentified”, and their characteristic parameters were recorded. The reliability of compound identification was ensured by assessing the reproducibility of the results through three parallel experiments.

### 2.5. Electronic Tongue Measurement

The samples analyzed in this experiment were unfermented pomegranate juice (control group) and pomegranate juice samples after mixed-culture fermentation. The actual sample preparation steps were as follows: each group consisted of 25 replicates, 30 mL from each sample was placed into a 50 mL beaker and used directly for electronic tongue detection, without any additional preprocessing (such as centrifugation or filtration). Measurements were performed using an electronic tongue (Model 402B-C; Beijing Invo Way Technology Development Co., Ltd., Beijing, China) equipped with eight taste sensor electrodes: sourness, bitterness, astringency, aftertaste B, aftertaste A, umami, richness, and saltiness. The detection parameters were set as follows: measurement time was 120 s (to ensure that the sensors fully reacted with the taste substances in the sample and stable response values were obtained), and the sensor rinsing time was 10 s (electrodes were washed with a special cleaning solution to eliminate interference from residual taste substances from previous samples). A two-step cleaning method was used to activate and calibrate the sensors during testing to ensure the detection accuracy. Ultimately, the average of the last three stable response signals of each sample group was used as the final detection result to improve data repeatability [17].

### 2.6. Non-Targeted Metabolomic Analysis

The samples were sent to Majorbio Bio-Pharm Technology Co., Ltd. (Majorbio Bio-Pharm Technology Co., Ltd., Shanghai, China) for analysis under dry ice conditions. Six biological replicates of sour pomegranate juice were collected before fermentation and four after fermentation. Approximately 200 ± 5 mg of each sample was transferred into a 2 mL centrifuge tube, to which one grinding bead with a diameter of 6 mm and 800 µL of extraction solution (methanol: water = 4:1, *v*/*v*) were added. The extraction solution contained four internal standards (0.02 mg/mL L-2-chlorophenylalanine). The samples were ground using a tissue grinder at −10 °C and 50 Hz for 6 min, followed by ultrasonic extraction at 5 °C and 40 KHz for 30 min. After centrifugation for 15 min (13,000× *g*, 4 °C), the supernatant was transferred to sample vials for instrumental analysis. Simultaneously, 20 µL of supernatant from each sample was combined and used as a quality control (QC) sample.

Analysis was performed using an UHPLC-Q Exactive system (UHPLC-Q Exactive; Thermo Fisher Scientific Inc., Waltham, MA, USA). The chromatographic column was an ACQUITY UPLC BEH C18 (100 mm × 2.1 mm i.d., 1.7 µm; Waters Corporation, Milford, MA, USA); mobile phase A was 2% acetonitrile in water (containing 0.1% formic acid), and mobile phase B was acetonitrile (containing 0.1% formic acid). The injection volume was 3 μL, and the column temperature was set at 40 °C. The mass spectrometry signals of the samples were acquired using both positive and negative ion scanning modes, with an m/z range of 70–1050. The spray voltage in positive ion mode was 3500 V, and in negative ion mode was −3000 V; sheath gas was set at 50 arb, auxiliary heating gas at 13 arb, ion source temperature at 450 °C, and the stepped collision energy was 20, 40 and 60 V [18].The gradient elution program was set as follows, with a flow rate of 0.3 mL/min: 0.0–2.0 min, 95% mobile phase A and 5% mobile phase B (eluting highly polar metabolites); 2.0–8.0 min, mobile phase A linearly decreased from 80% to 50%, and mobile phase B linearly increased from 20% to 50% (eluting moderately polar metabolites); 8.0–12.0 min, mobile phase A linearly decreased from 50% to 5%, and mobile phase B linearly increased from 50% to 95% (eluting weakly polar to non-polar metabolites); 12.0–14.0 min, maintained at 5% mobile phase A and 95% mobile phase B (washing the column and removing residual metabolites); 14.0–14.1 min, rapidly returned to 95% mobile phase A and 5% mobile phase B; 14.1–17.0 min, maintained at 95% mobile phase A and 5% mobile phase B (equilibrating the column in preparation for the next sample analysis). This gradient elution program, combined with the above flow rate, ensured comprehensive coverage and effective separation of the 1309 metabolites identified in the pomegranate juice samples.

After the raw LC-MS data were processed using Progenesis QI software to generate a data matrix, they were uploaded to the corresponding metabolomics cloud analysis platform. During data preprocessing, missing values were first filtered using the 80% rule (metabolites missing in more than 20% of any group were excluded), and the remaining missing values were filled using the group median. Total peak area normalization was then performed to eliminate sample concentration differences and instrument fluctuation errors. Finally, variables with a relative standard deviation (RSD) greater than 30% in the QC samples were removed to ensure data reliability. Principal component analysis (PCA) and orthogonal partial least squares discriminant analysis (OPLS-DA) were conducted using the R package “ropls” (Version 1.32.0). Model stability was assessed through 7-fold cross-validation (requiring model explanatory power R^2^Y > 0.9 and predictive ability Q^2^ > 0.8) and 200 permutation tests (requiring the intercept of the Q^2^ regression line with the Y-axis to be less than 0.05, to avoid model overfitting). Subsequently, based on the differential metabolite set “Fermented_vs_Control”, a total of 126 differential metabolites were identified (screened using the combined criteria of “VIP ≥ 1, *p* < 0.05, FC ≠ 1”); then, according to the KEGG annotation rate in the metabolite overview, metabolites that could not be matched to a KEGG Compound ID were removed from these 126 differential metabolites, resulting in 89 differential metabolites available for KEGG pathway enrichment analysis. After standard screening, a total of 1309 metabolites from the background metabolite set (Set_Origin) constructed after data preprocessing were used as background. These originated from the original detected metabolite set “Set_Raw” (which contained 1314 metabolites). After processes such as filtering for missing values (80% rule), QC sample RSD screening (removing metabolites with RSD > 30%), and data normalization, the set of “metabolites with clear name annotations” was selected, ultimately including 1309 metabolites in the analysis. Pathway enrichment analysis was performed using Fisher’s exact test (with a significance threshold of adjusted *p* < 0.05). The enrichment factor is calculated as “the number of differential metabolites enriched in a pathway/the total number of metabolites annotated for that pathway,” ultimately revealing the biological pathways involved in the differential metabolites [19].

### 2.7. Statistical Analysis

Statistical analyses were performed using SPSS software (version 26.0; International Business Machines Corporation, Armonk, NY, USA). A one-way analysis of variance (ANOVA) was conducted, followed by Duncan’s multiple range test for multiple comparisons to determine significant differences. The threshold for statistical significance was set at *p* < 0.05. Origin 9.1 software (OriginLab Corporation, Northampton, MA, USA) was used to fit the linear and nonlinear equations for analysis.

## 3. Results and Discussion

### 3.1. Optimal Fermentation Conditions

#### 3.1.1. Screening of Fermentation Strains

Figures S1–S5 shows the total acid content continuously increased with the extension of fermentation time, which is highly consistent with the typical characteristic of acid production during lactic acid bacteria fermentation and metabolism. Lactic acid bacteria utilize fermentation substrates and continuously generate organic acids, such as lactic acid, through glycolysis and other metabolic pathways [20]. There were obvious differences in the curves corresponding to different inoculation amounts, indicating that the inoculation amount had a significant regulatory effect on the acid production rate of lactic acid bacteria and the accumulation of total acid. In the late stage of fermentation, owing to the combined influence of substrate limitation and other factors, the accumulation of total acid under different inoculation amounts tends to stabilize [21].

The TSS content did not change significantly throughout the fermentation cycle. Soluble solids are mainly composed of sugars and other substances; however, in this experimental system, the dissolution and transformation of other substances may have balanced out the consumption of sugars. Alternatively, within the range of inoculum amounts and fermentation durations set in this experiment, the consumption of sugars and changes in other components in the system may have offset each other, ultimately maintaining the relative stability of the soluble solids content [22].

This study determined the parameters for mixed-strain fermentation of pomegranate juice, specifically a strain ratio of 45% *L. fermentum*, 31% *L. plantarum*, and 28% *L. acidophilus*, with an inoculation amount of 2% and a fermentation time of 11 h. Combined analysis using an electronic tongue, electronic nose, and GC-IMS confirmed that fermentation significantly improved the sensory quality of sour pomegranate juice, resulting in a richer taste profile and a more complex and distinctive aroma. Untargeted metabolomics revealed that the contents of triterpenes, cyclic peptides, other alkaloids and their derivatives, oligopeptides, other steroids and their derivatives, dipeptides, glycosides, organic acids and their derivatives, macrolides and their derivatives, acyl lipids, alpha amino acids and their derivatives, nucleotides and their derivatives, as well as coumarins and their derivatives, all increased significantly after fermentation, suggesting that microbial metabolism drove the enrichment of these characteristic substances. These results provide scientific support for the precise optimization of food fermentation processes, flavor-oriented regulation, and the development of high-quality fermented foods from the perspective of metabolic changes in sensory phenotypes [23].

The polyphenol content shows a trend of first increased and then decreased, reaching its peak at approximately 12 h of fermentation. This phenomenon is closely related to the metabolic activation and substrate transformation activities of lactic acid bacteria during fermentation. In the early stages of fermentation, the metabolic activities of lactic acid bacteria are activated, prompting the transformation of polyphenol precursor substances in the fermentation substrate or breaking down the structure of plant cells, thereby facilitating the release of polyphenols [24]. In the later stages of fermentation, as metabolic products continue to accumulate and the activity of related enzymes changes, polyphenols undergo decomposition or transformation, leading to a decrease in their content [25]. Specifically, under a 3% inoculation level and 12 h of fermentation, the polyphenol content of *L*. *fermentum, L. casei*, *L. acidophilus*, and *L. plantarum* reached a peak, whereas for *L. rhamnosus*, the peak polyphenol content was observed at a 1% inoculation level and 12 h of fermentation. The order of polyphenol content was as follows: *L. fermentum* > *L. plantarum* > *L. acidophilus* > *L. casei* > *L. rhamnosus*. In summary, *L. fermentum*, *L. plantarum*, and *L. acidophilus* were selected for mixed-culture fermentation experiments.

#### 3.1.2. Screening of Mixed Bacteria Ratios

Figure S6A shows, after 12 h, the polyphenol content of group 8 reached 598.33 mg GAE/100 g FW, which was the highest among the eight groups. This may be because lactic acid bacteria produce enzymes, such as β-glucosidase, during fermentation, catalyzing the release of bound polyphenols from their glycosylated forms to free polyphenols. Simultaneously, microbial transformation or compound depolymerization promotes the production and accumulation of polyphenols [26]. In terms of total acid content (Figure S6B), Group 1 had the highest level. Notably, when the total acid content is within an appropriate range, it enhances the product’s flavor. However, once it exceeds the threshold, it can lead to increased sourness and reduced viscosity and may also cause flavor deterioration [27]. No significant changes were observed in the soluble solid content among the groups (Figure S6C), which is consistent with previous studies [28]. Based on these results, a mixed-strain ratio of 45% *L. fermentum*, 31% *L. plantarum*, and 28% *L. acidophilus* was recommended.

#### 3.1.3. Screening of Mixed-Strain Inoculation Amount

**Figure 1:**
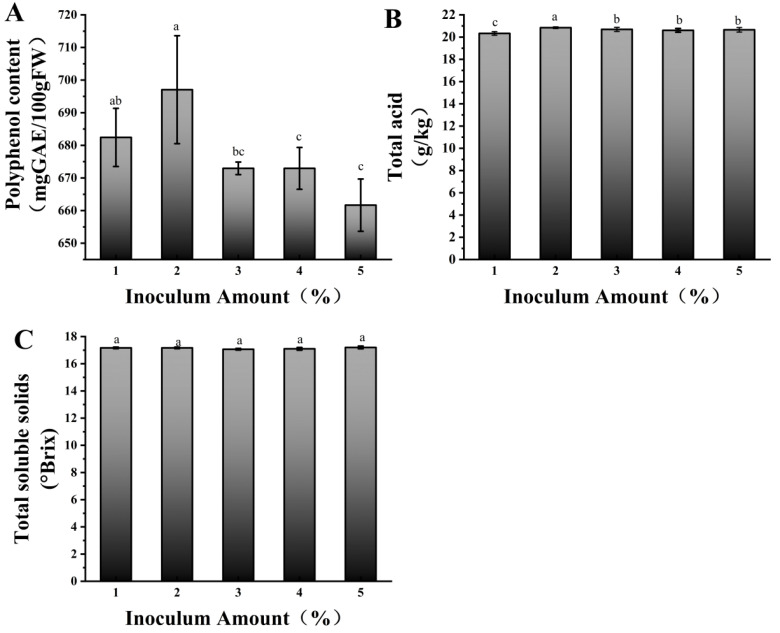
Polyphenol content (**A**), total acid content (**B**), and soluble solids (**C**) at 12 h of fermentation with different mixed inoculation amounts. Different lowercase letters indicate significant differences (*p* < 0.05).

We further optimized the fermentation time and inoculation amount for the mixed-strain fermentation. Figure 1A shows, after 12 h of fermentation, the polyphenol content varied significantly with different inoculation amounts, reaching its highest value at 2% inoculation, which was significantly higher than that of the other treatments. The lowest polyphenol content was observed at 5% inoculation level. This is because when the inoculation amount is too high, the growth and metabolism of lactic acid bacteria changes. On one hand, a large number of lactic acid bacteria rapidly consume nutrients in the fermentation system, leading to insufficient energy and substrate supply for polyphenol metabolism [29]. Figure 1B shows the total acid content was highest at 2% inoculation and lowest at 1%, indicating that a moderate increase in inoculation can promote acid production, but further increasing the inoculation amount does not enhance the acid content. The acid production by lactic acid bacteria is influenced by substrate concentration and population density. At low inoculation amounts, bacterial proliferation is slow, and the acid production rate is low. A moderate inoculation amount promotes rapid substrate utilization and acid production. At high inoculation amounts, the substrate is consumed too quickly, and the accumulation of metabolic by-products (such as organic acids) inhibits bacterial growth, resulting in stabilized acid production [30]. No obvious difference was observed in the TSS content corresponding to different mixed-strain inoculation levels (Figure 1C). In summary, 2% was the inoculation volume for mixed-strain fermentation.

#### 3.1.4. Screening of Mixed-Strain Fermentation Time

**Figure 2:**
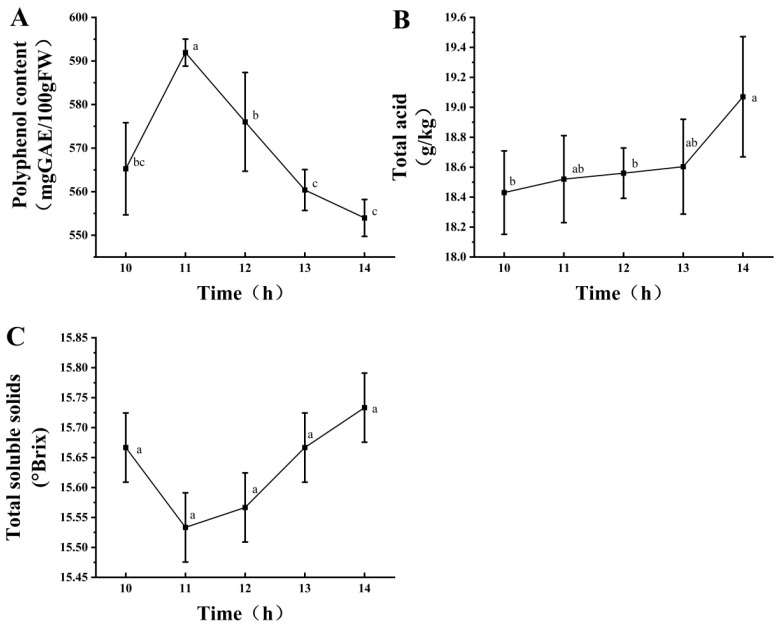
Effects of different fermentation times at a 2% inoculation level on polyphenol content (**A**), total acid content (**B**), and soluble solids (**C**). Different lowercase letters indicate significant differences (*p* < 0.05).

Figure 2 shows under a fixed mixed-strain inoculum of 2%, the polyphenol content initially increased and then decreased, reaching its highest value at 11 h (Figure 2A). The total acid content showed an upward trend with the extension of fermentation time, reaching its peak at 14 h (Figure 2B), whereas the TSS content showed no significant change (Figure 2C). Therefore, the fermentation time was 11 h. In summary, the conditions for mixed-strain fermentation were as follows: fermentation time, 11 h; inoculation amount, 2%; and strain ratio, 45% *L. fermentum*, 31% *L. plantarum*, and 28% *L. acidophilus*. These conditions were also used for sample preparation for subsequent analyses.

### 3.2. Impact of Fermentation on Flavor Compounds in Sour Pomegranate Juice

#### 3.2.1. Electronic Nose and Analysis

**Figure 3:**
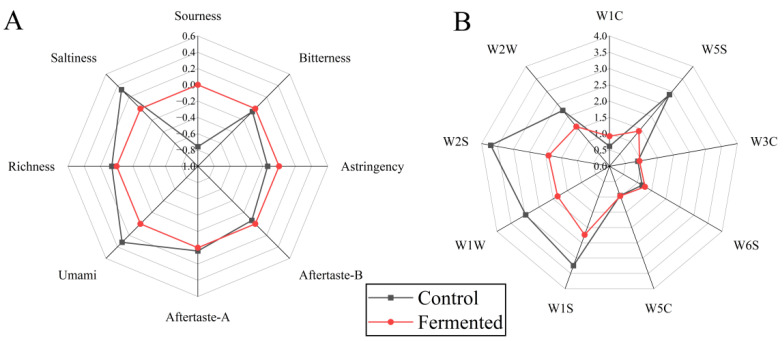
Electronic tongue (**A**) and electronic nose (**B**) analysis of pomegranate juice samples before and after fermentation. Note: The sensors correspond to different types of gases or chemicals, respectively, W1C, aromatic components and benzene; W5S, nitrogen oxides; W3C, aromatic components and ammonia substances; W6S, hydrogen; W5C, hydrogen compounds; W1S, methyl substances; W1W, sulfides; W2S, alcohols, aldehydes and ketones; W2W, aromatic components and organic sulfide.

Figure 3A shows (Electronic nose analysis), the Fermented group exhibited significantly higher responses in dimensions such as W1C, W2S, W1S, and W5C compared to the Control group, indicating that fermentation promotes an increase in volatile compounds such as alcohols and aldehydes (e.g., hexanal, nonanal), terpenes (e.g., limonene), and sulfur-containing compounds (e.g., methanethiol). Among these, substances such as esters and aldehydes contribute to the aroma, while alcohols impart a mellow texture and rich flavor [31]. The Control group showed only slightly higher responses in a few dimensions, such as W5S (nitrogen oxides associated with faint, irritating odors), with overall flavor compounds being sparse.

Figure 3B shows (Electronic tongue analysis), the nine characteristics of pomegranate juice displayed significant differences before and after fermentation. The radar chart for Fermented extends noticeably in the dimensions of umami, richness, and Aftertaste-A, indicating that fermentation increases the content of umami substances (such as amino acids), thereby enhancing the mouthfeel and persistence of positive aftertaste. In addition, its salty and astringent dimensions are prominent, which may be due to changes in inorganic salt metabolism and polyphenol transformation caused by fermentation [32]. In contrast, the control sample had more pronounced sourness, bitterness, and Aftertaste-B, suggesting that the unfermented sample retained more of the basic sour and bitter components, resulting in a more stimulating taste profile.

#### 3.2.2. GC-IMS Analysis

##### Three-Dimensional Comparative Analysis of the Ion Mobility Spectra of Volatile Components in Samples Before and After Fermentation

**Figure 4:**
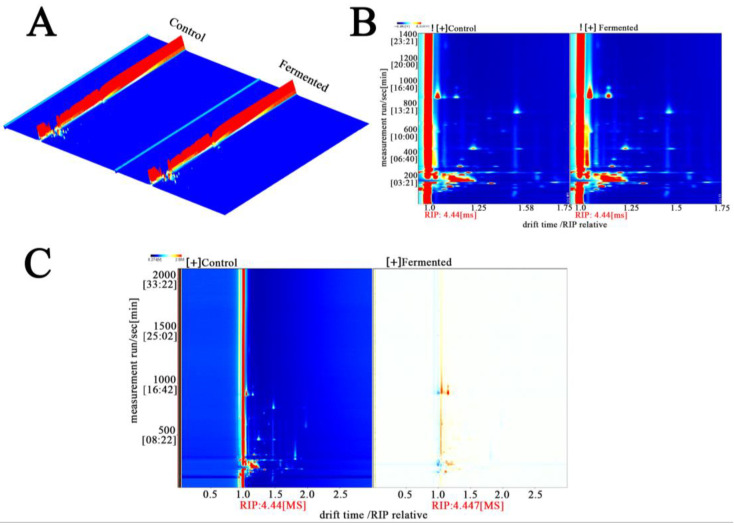
Three-dimensional differential ion mobility spectra (**A**), 2D ion mobility spectra (**B**), and 2D MASS spectrometry (**C**) analysis of metabolomic fingerprints in sour pomegranate juice samples before and after fermentation. Note: In the plots, the color gradients (from blue to red) indicate the signal intensity (red/orange regions correspond to higher metabolite ion/mass signals). In panel A, the red regions represent the control group, and the orange-red regions represent the fermented group (as labeled); panels B and C use color gradients to illustrate the differential ion/mass signal distributions between the Control and Fermented samples. “RIP” denotes the reactant ion peak, which serves as a reference for drift time normalization.

Figure 4A shows GC-IMS detection generates three-dimensional data on retention time (chromatographic separation), drift time (ion mobility separation), and peak intensity (component concentration). The 3D diagram accurately presents these characteristics: retention time reflects the separation of VOCs within the chromatographic column owing to differences in boiling point and polarity, whereas drift time is determined by their molecular structure (size and charge characteristics). Together, these two parameters serve as core qualitative indicators. The peak intensity, represented by color and height, indicates the concentration. The prominent red regions denote high-VOC content, which constitutes the fundamental substances underlying the flavor of sour pomegranate juice.

Figure 4B shows this figure projects three-dimensional data into a two-dimensional top view and uses colors to represent peak intensity (red indicates high intensity, blue indicates low intensity), a method that simplifies the observation dimensions. From the figure, the distribution characteristics of volatile organic compounds (VOCs) in sour pomegranate juice can be macroscopically distinguished: in the unfermented sample (Control), there are dense red peaks within a specific drift time range (e.g., 1000–1200 s), which indicates that this range is rich in initial VOCs (esters, aldehydes, and other compounds with fruity/grassy flavors); in the fermented sample (Fermented), new red peaks appear within a new drift time range (e.g., 800–1000 s), which shows that the fermentation process promotes the formation of new VOCs and provides a material basis for the change in aroma [33].

Figure 4C shows the difference map amplifies the changes in VOCs caused by fermentation by comparing the fermented and unfermented samples. The blue/dark regions correspond to components that are “unique to or present in higher amounts in the Control,” most of which are natural background flavor substances in pomegranate juice (such as certain alcohols and aldehydes) that are consumed or transformed by microbial metabolism during fermentation. The yellow regions represent components that are unique to, or present in higher concentrations in, the fermented sample. These compounds—primarily microbial metabolites formed during fermentation, such as characteristic esters and furans—introduce new layers of flavor to the pomegranate juice [34].

##### PCA of Sour Pomegranate Juice Samples

**Figure 5:**
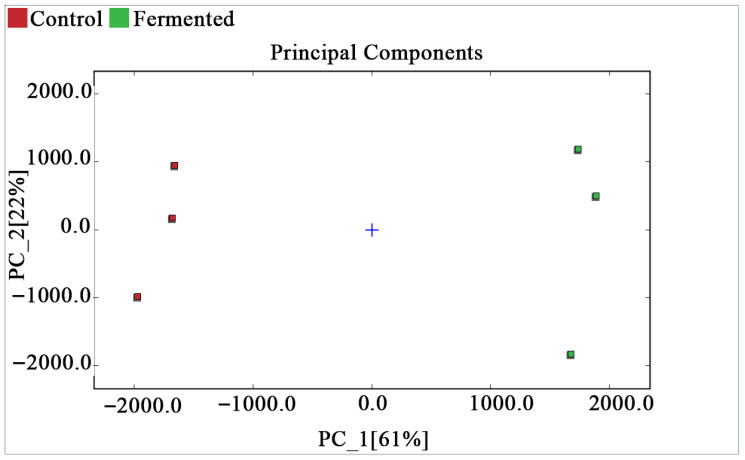
PCA score plot of pomegranate juice samples.

Figure 5 shows the PCA score plot, which presents the distribution of each sample in the principal component space. Based on the volatile organic compounds (VOCs) data characteristics of pomegranate juice and the Dynamic PCA plugin, the analysis focused on three key parameters to ensure reliability: during data preprocessing, the plugin automatically standardizes the original peak intensity data of VOCs (eliminating dimensional bias) and removes non-informative variables with zero variance; principal components are extracted based on a cumulative variance contribution rate of ≥70%. In this study, PC_1 (61%) and PC_2 (22%) had a cumulative contribution rate of 83%; therefore, both were selected for further analysis. Model reliability was verified using the plugin’s default 95% confidence ellipse, and no outliers were found among all samples, indicating good data reproducibility and a reliable model. The blue cross in the center of the plot represents the centroid of all samples. The samples of the control group (red squares) and the fermented group (green squares) are distributed with significant differences along the PC_1 axis and relatively similar distributions along the PC_2 axis. This confirms that, combined with PCA, the differences in volatile organic compounds can effectively distinguish and classify the two types of samples.

##### Fingerprint Profile of Volatile Organic Compounds in Pomegranate Juice Samples Before and After Fermentation

**Figure 6:**
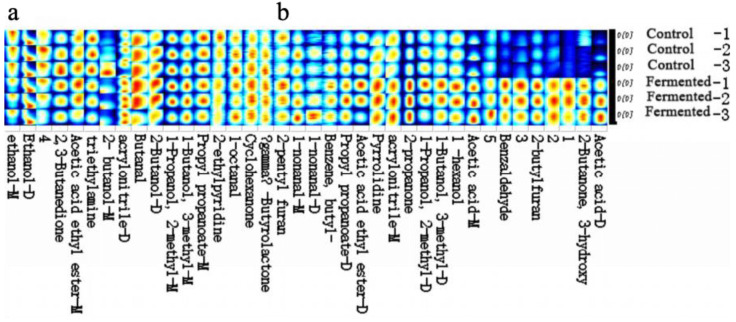
Gallery plot (fingerprint profile) of the pomegranate juice samples before and after fermentation. Note: “−1, −2, −3” represent three biological replicates for each group. Part (**a**) and part (**b**) respectively display distinct segments of the volatile compound fingerprint profiles, with each segment corresponding to different identified volatile metabolites in the pomegranate juice samples. The color gradient (from blue to yellow/red) indicates the relative signal intensity or content of each volatile metabolite, where blue represents lower intensity/content and red or yellow represents higher intensity/content.

Figure 6 presents the gallery plot displaying the volatile compound fingerprints of the pomegranate juice samples. “Control-1, -2, -3” and “Fermented-1, -2, -3” represent three parallel replicates for the control and fermented groups, respectively. GC-IMS was employed to examine the differences in volatile organic compounds (VOCs) of pomegranate juice before and after fermentation. Table S1 lists 38 identified compounds, including nine alcohols, four ketones, five aldehydes, four esters, two acids, five nitrogenous compounds, two furans, one lactone, one aromatic hydrocarbon, and five unidentified volatile compounds. Table 1 summarizes the aroma components whose levels were either enhanced or reduced after fermentation.

To visualize the variation patterns and distribution characteristics of volatile compounds, the Gallery Plot function of the GC-IMS software was used to generate a fingerprint spectrum. Figure 6 reveals marked differences in both the types and concentrations of compounds before and after fermentation (as indicated by the intensity of the spectrum colors). Samples with brighter colors correspond to stronger aroma intensities, suggesting a stratified distribution of volatile substances.

The fingerprint spectrum was divided into two regions, a and b, based on the central wavelength. Volatile compounds in panel a were detected in both pre- and post-fermentation samples, whereas those in panel b represent key flavor-forming components, including alcohols and compounds contributing to creamy notes—typical metabolic products of microbial sugar fermentation. Consequently, fermentation promotes the formation of alcohols, imparting characteristic fermented aromas [35].

Region b, characterized by higher concentrations of specific substances in the fermented samples, forms the core of their distinctive flavor profile. Among these, esters enhance fruity aromas, benzaldehyde and 2-butylfuran contribute unique complexity, while short-chain alcohols and low-molecular-weight acids impart fresh green notes and a mild acidic tang [14]. Table 1 further indicates that the majority of VOCs exhibited an upward trend after fermentation, suggesting that microbial metabolism (e.g., by yeasts and lactic acid bacteria) facilitates the formation or accumulation of volatile compounds, thereby directly shaping the flavor characteristics of pomegranate juice [36].

### 3.3. The Impact of Mixed-Strain Fermentation on the Metabolic Profile of Pomegranate Juice

#### 3.3.1. PLS-DA/OPLS-DA Score Plots and Permutation Tests

The experimental data used for the Partial Least Squares Discriminant Analysis (PLS-DA) and permutation test in this section were derived from the volatile organic compounds (VOCs) detection data of sour pomegranate juice samples, acquired by the Flavor Spec^1^H^1^-00053 GC-IMS. Specifically, the data included the peak volume (representing relative content) of each identified VOC in unfermented sour pomegranate juice samples, fermented sour pomegranate juice samples, and the peak volume data of QC samples.

**Figure 7:**
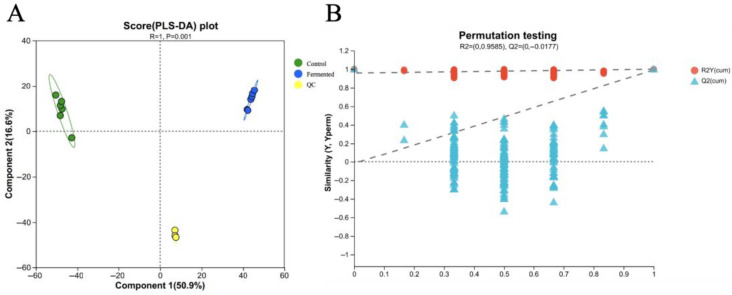
PLS-DA score plot (**A**) and permutation test plot (**B**) for the metabolomic profiles of sour pomegranate juice (Control, Fermented, and QC) before and after fermentation. Note: In plot A, green circles represent control samples, blue circles represent fermented samples, and yellow circles represent QC (quality control) samples. In plot B, the dashed lines denote the reference thresholds for cumulative R^2^ (red, R2Y(cum)) and cumulative Q^2^ (blue, Q2Y(cum)) in permutation testing, which were used to evaluate whether the PLS-DA model was overfitted.

Figure 7A shows the score plot of Partial Least Squares Discriminant Analysis (PLS-DA). It can be observed from the figure that the unfermented pomegranate juice samples (Control) and the fermented pomegranate juice samples (Fermented) are significantly separated along the first principal component (Component 1, explaining 50.9% of the variation). The determination coefficient of the PLS-DA model, R^2^Y (cum) = 0.978, and the predictive coefficient, Q^2^ = 0.786, indicate that the impact of fermentation on the metabolome of pomegranate juice is significant and that the model demonstrates a good fit and predictive ability, making the results reliable. In addition, QC samples are tightly clustered in the plot, further validating the stability and reproducibility of the entire experimental process. These results suggest that fermentation can regulate the composition of metabolites in pomegranate juice, resulting in metabolite profiles with clearly distinguishable differences [36].

Figure 7B shows a permutation test plot for validating the Orthogonal Partial Least Squares Discriminant Analysis (OPLS-DA) model. This plot illustrates the distribution of R^2^Y(cum) (represented by red dots) and Q^2^(cum) (represented by blue triangles) after the data permutation. With R^2^Y(cum) = 0.9585 and Q^2^(cum) = −0.0177, the permutation test results indicated that the performance of the original OPLS-DA model was significantly superior to that of the permuted models, demonstrating that the model does not suffer from overfitting. Thus, the OPLS-DA model can reliably and accurately distinguish metabolic differences in sour pomegranate juice induced by fermentation, laying a solid foundation for subsequent differential metabolite screening.

Given that VOCs data are characterized by “high dimensionality and strong collinearity” (for example, different VOCs may originate from the same metabolic pathway or be influenced by the same experimental conditions), it is difficult to fully distinguish “systematic variation related to grouping” from “random within-group nois” (such as minor physiological differences between samples or slight fluctuations in instrument detection) by using PLS-DA alone. Therefore, this study incorporated the OPLS-DA model, which decomposes the original data into “predictive principal components related to grouping” and “orthogonal components unrelated to grouping”, thereby effectively eliminating the noise.

Compared to PLS-DA, OPLS-DA has two main advantages: first, it enhances the clarity of separation between groups, especially for VOCs components that are “low in abundance but high in biological significance” (such as trace compounds influencing flavor), reducing noise interference to accurately pinpoint differential signals; second, it simplifies model interpretation by removing irrelevant variations, allowing direct focus on the causal relationship between “fermentation and VOCs changes,” and avoiding deviations in conclusions due to noise superposition. This forms a progressive analytical logic: “PLS-DA validates the existence of overall differences→OPLS-DA precisely identifies key differential signals”—with PLS-DA confirming the presence of significant overall differences in VOCs before and after fermentation (model R^2^Y and Q^2^ close to 1, permutation test *p* < 0.05, meeting reliability requirements), and OPLS-DA enabling precise screening of key differential VOCs, fully aligning with the core objective of “analyzing the mechanism by which fermentation regulates the VOCs flavor profile in pomegranate juice.” Based on the PLS-DA model above, this study further calculated the variable importance in projection (VIP) score for each VOC, using VIP ≥ 1 as the screening threshold for key differential metabolites, to evaluate the contribution of different VOCs to the separation between groups.

#### 3.3.2. Differential Metabolite Analysis

Table S3 shows these 126 identified compounds—screened with a fold change (FC) > 1.5—were categorized into primary substance classes based on conventional classification criteria, as follows: others (41), lipids (18), flavonoids (14), phenolic acids and their derivatives (11), amino acids and their derivatives (10), terpenoids (7), carbohydrates and their derivatives (7), organic acids and their derivatives (6), steroids and their derivatives (5), alkaloids and their derivatives (2), nucleotides and their derivatives (2), coumarins and their derivatives (1), indoles and their derivatives (1), and quinones (1). Table 2 summarizes the metabolomic analysis results obtained from six unfermented (control) and four fermented pomegranate juice samples. All samples were analyzed using GC-IMS, and metabolite levels were expressed as peak volumes (relative abundances). The column “Categories of Metabolites” is defined according to biochemical structure or biosynthetic pathway, whereas “Metabolite” refers to the specifically identified compounds. The “*p*-value” indicates the statistical significance of the differences between groups (all *p* < 0.05, confirming the reliability of the observed differences). “FC (Fold Change)” represents the ratio of metabolite abundances between groups (all FC > 1.5, indicating significant upregulation after fermentation). The category “Other” mainly includes oligopeptides, suggesting that fermentation promotes peptide formation.

Triterpenoids possess important physiological functions such as antioxidant and anti-inflammatory effects. Among them, the FC value of Presqualene diphosphate is the highest of all substances. As a key precursor for the synthesis of squalene, cholesterol, and other triterpenoid compounds, a significant increase in its content is of great importance. Squalene is a core component in maintaining the integrity of the skin barrier, while cholesterol serves as a fundamental raw material for cell membrane construction and hormone (such as sex hormones and adrenocorticosteroids) synthesis. This substantial increase indicates that the fermentation process significantly activates the triterpenoid synthesis pathway [37]. Furthermore, ganoderic acid H, a triterpenoid specifically produced by *Ganoderma* spp., was detected in pomegranate juice samples. The occurrence of this compound in the juice is presumably associated with residual viable fungal cells (e.g., those belonging to the genus Ganoderma) that survive standard sterilization processes—complete microbial elimination remains technically challenging in such matrices. We hypothesize that the initial *Lactobacillus* spp.-dominated fermentation may establish a favorable microenvironment, potentially via acid-catalyzed hydrolysis of precursor substances. This hypothesis is corroborated by existing evidence demonstrating that lactic acid fermentation can lower the system pH and promote the hydrolysis of glycosylated triterpene precursors, thereby enabling these residual fungal contaminants to biotransform endogenous substrates into ganoderic acid H. Notably, this biotransformation is regarded as a secondary event, as the primary microbial and biochemical alterations—encompassing rapid pH decline, lactic acid accumulation, and a marked increase in *lactobacilli* viability—are unequivocally driven by the inoculated bacteria. This finding is consistent with extensive literature confirming *lactobacilli* as the principal mediators of lactic acid fermentation processes [38].

Among peptide substances, Nummularine A is the most abundantly increased component within the category of cyclic peptides. Owing to their unique cyclic structure, cyclic peptides possess enhanced physicochemical stability. They not only provide the body with essential amino acids, such as valine and leucine, but may also have potential immunomodulatory activity. After fermentation, both their nutritional supply capacity and functional properties are significantly improved [39].

Mucronine D, on the other hand, is an oligopeptide that, thanks to its high efficiency of intestinal absorption, accumulates in large amounts after fermentation and can rapidly replenish amino acids for the body. This makes it particularly suitable for individuals with weaker digestive function [40].

In addition, Arg-Val-Phe and Met-Lys-Lys are both typical tripeptides; the former consists of arginine, valine, and phenylalanine. Arginine participates in immune regulation and vasodilation, valine helps maintain muscle function, and phenylalanine is a key precursor of neurotransmitter synthesis. The latter contains methionine and two lysine residues, respectively. Methionine, a sulfur-containing essential amino acid, is involved in protein synthesis and methyl transfer reactions, whereas lysine promotes calcium absorption and collagen synthesis. The significant accumulation of these two tripeptides after fermentation provides the body with a precise supply of multiple essential amino acids [41].

Fermentation-induced changes in steroids and their derivatives are also noteworthy. The mechanism of action of cimiracemoside D is similar to that of phytosterols, as it regulates metabolism by competitively inhibiting cholesterol absorption. After fermentation, its content doubles, which plays a positive role in maintaining stable serum lipid levels. As a major component of bile acids, taurocholic acid’s core function is to emulsify fats in the intestine, facilitating better contact between lipase and fats, thereby improving the digestion and absorption efficiency of fats and fat-soluble vitamins. The increase in its content after fermentation directly enhances the body’s ability to utilize lipid nutrients [42].

#### 3.3.3. KEGG Pathway Enrichment Analysis

**Figure 8:**
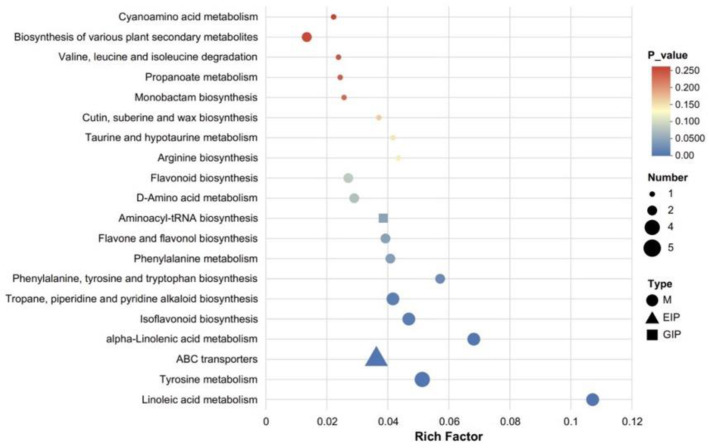
KEGG pathway enrichment analysis of differential metabolites identified between fermented and control pomegranate juice samples (Fermented_vs_Control), with the background comprising “Set_Origin” and 1309 annotated metabolites. The bubble chart displays the top enriched metabolic pathways. The x-axis represents the Rich Factor, defined as the ratio of the number of differential metabolites annotated in a given pathway to the total number of annotated metabolites in that pathway. The size of each bubble corresponds to the number of differential metabolites involved, while the color gradient indicates the statistical significance (*p*-value), with darker red representing higher significance. Different shapes denote metabolite classes: circles (M), triangles (EIP), and squares (GIP).

Figure 8 presents the KEGG pathway enrichment bubble plot (Fermented_vs_Control). Using the “Set_Origin” dataset containing 1309 annotated metabolites as the background, this plot illustrates intergroup differences according to the x-axis (enrichment ratio), y-axis (pathway name), bubble color (FDR value), and bubble size (number of differentially expressed metabolites). Among these pathways, “Linoleic acid metabolism” shows the highest enrichment ratio (≈0.11) with FDR < 0.05 (blue bubble), related to microbial degradation of unsaturated fatty acids. “Tyrosine metabolism” exhibits an enrichment ratio of ≈0.055 with FDR < 0.05; amino acid derivatives from this pathway may indirectly support alkaloid synthesis by providing intermediate metabolites. Pathways such as “Phenylalanine metabolism” and “Flavonoid and flavonol biosynthesis” also display notable enrichment (FDR < 0.1), associated with the upregulation of glycosides, coumarin derivatives, and other compounds involved in flavor and antioxidant component accumulation. In contrast, pathways like “Cyanogenic amino acid metabolism” are not significantly enriched (FDR > 0.1), suggesting that fermentation has little effect on these processes. Overall, the results indicate that fermentation drives metabolite alterations primarily by activating fatty acid, amino acid, and secondary metabolite pathways, consistent with the observed differential metabolite profiles and providing pathway-level evidence elucidating the regulatory mechanisms underlying fermentation [43].

## 4. Conclusions

This study aimed to optimize the mixed-strain fermentation process of pomegranate juice and elucidate the mechanisms through which fermentation enhances its quality, thereby providing a scientific basis for the development of high-quality fermented pomegranate juice products. The parameters for mixed-strain fermentation were as follows: a strain ratio of 45% *L. fermentum*, 31% *L. plantarum*, and 28% *L. acidophilus*; an inoculation amount of 2%; and a fermentation time of 11 h. Subsequently, a combined analysis using an electronic tongue, electronic nose, and GC-IMS confirmed that fermentation significantly improved the sensory quality of the sour pomegranate juice, specifically enhancing taste richness and forming a more complex, distinctive aroma. This directly addresses the core demand for enhanced sensory quality in the processing of fermented sour pomegranate juice. Moreover, untargeted metabolomics revealed that the levels of functional and flavor-related substances (including triterpenes, cyclic peptides, other alkaloids and their derivatives, oligopeptides, other steroids and their derivatives, dipeptides, glycosides, organic acids and their derivatives, macrolides and their derivatives, acyl lipids, alpha amino acids and their derivatives, nucleotides and their derivatives, and coumarins and their derivatives) increased significantly after fermentation. This finding clarifies that microbial metabolism is the key driver of the enrichment of these characteristic substances, revealing the material basis for fermentation-induced quality improvement in sour pomegranate juice. Ultimately, these results offer scientific support for the precise optimization of food fermentation processes and flavor-oriented regulation, and provide a theoretical reference for the development of high-quality fermented foods from the perspective of the link between sensory phenotype changes and metabolic shifts.

## 5. Limitations and Prospects of Research

As shown in Table 2, the control group in the fermentation experiment included six biological replicates, whereas the fermentation group had only four biological replicates, resulting in a relatively small overall sample size. This may lead to insufficient detection of differential signals in low-abundance metabolites. Although the reliability of the PLS-DA and OPLS-DA models in Figure 7 was verified through 200 permutation tests (*p* < 0.05), metabolomics data were characterized by high dimensionality (a total of 1314 metabolites were identified), which poses a risk of overfitting due to the “more variables than samples”.

Two core challenges must be addressed to advance future research. First, there is the difficulty of integrating the multidimensional data. If subsequent experimental groups are added with different fermentation parameters (such as strains or duration), vast amounts of intertwined GC-IMS volatile compound data and metabolomic data will be generated. Efficiently correlating these two types of data and excluding irrelevant interference signals places higher demands on the accuracy and integration capability of analytical methods. Second, there are technical barriers to verifying the functionality and stability of these systems. The new flavor compounds and functional metabolites produced after fermentation require confirmation of their stability during storage, as well as their direct relationships to product palatability and functionality. Such confirmation necessitates complex sensory evaluation experiments, long-term storage monitoring, and in vitro activity validation, all of which entail high experimental design and execution costs.

## Figures and Tables

**Table 1:** Changes in volatile compounds and their associated aroma attributes after fermentation.

Compound	Peak Volume Change (AU)	Peak Volume Change Rate (%)	Aromatic Attributes
Acetic acid-D	1469.64	217.08	Aromatic
Benzaldehyde	201.77	146.15	Pungent taste
2-Butanone, 3-hydroxy	24.44	122.96	Bitter almond flavor
Acetic acid-M	3458.88	77.41	Creamy aroma
Propyl propanoate-M	0.46	0.06	
Butanal	−28.92	−8.21	
2-Butylfuran	71.03	76.92	Irritating taste
2-propanone	1669.45	55.35	Sweet and spicy aroma (similar to fruit wine)
Pyrrolidine	110.36	41.96	Fruity and ether aroma
Propyl propanoate-D	36.49	26.4	Ammonia-like odor
1-Butanol,3-methyl-D	40.31	20.86	Fruity aroma
1-Hexanol	13.67	19.55	Alcohol, ether, and banana aromas
Benzene, butyl-	3.30	18	Fresh green, alcohol, fruit, and faint fatty aroma
Acrylonitrile-D	140.99	17.75	Slightly sweet and delicate fragrance
1-Nonanal-D	5.76	16.97	Pungent odor
Ethanol-M	−370.08	−19.51	
1-Nonanal-M	58.90	11.39	
1-Butanol,3-methyl M	120.83	15.68	Wax, citrus, fatty, and floral notes
Acrylonitrile-M	74.21	13.89	Mellow, ether, and banana aromas
1-Propanol,2-methyl-D	12.51	11.28	Wax, citrus, fatty, and floral aromas
Ethanol-D	1386.41	9.6	Distinct odor
2-Butanol-D	22.50	9.34	Alcohol odor
Acetic acid ethyl ester-D	107.92	8.85	Hint of mint
2-Butanol-M	7.67	7.58	Fruity aroma
2-Ethylpyridine	2.25	5.18	Minty fragrance
2-Pentyl furan	1.97	4.43	Green grass flavor
γ-Butyrolactone	1.18	0.45	Fruity, earthy, and potato-like scent
1-Propanol,2-methyl-M	−1.13	−0.11	Fruity aroma
1-Octanal	−6.12	−5.49	
Acetic acid ethyl ester-M	−2.50	−2.36	Distinctive odor
2,3-Butanedione	−115.96	−8.15	Rich and waxy fragrance
Cyclohexanone	−3.38	−10.9	Fruity and green leaf scent
Triethylamine	−62.18	−16.81	Minty and acetone-like odor

**Table 2:** Significantly upregulated metabolites and their classification, *p*-values, and fold change (FC) after sour pomegranate juice fermentation.

Categories of Metabolites	Metabolite	*p*_Value	FC (Fermented/Control)
Triterpenoid	Presqualene diphosphate	3.94 × 10^−0.7^	2.6877
	Lucidenic acid H	1.41 × 10^−14^	1.6145
Cyclic peptide	Nummularine A	7.48 × 10^−15^	2.6017
Other categories	Arg-Val-Phe	2.36 × 10^−9^	2.402
	Met-Lys-Lys	1.23 × 10^−8^	2.2977
	Thr-Pro	5.88 × 10^−6^	1.8648
	Glu-Leu-Ser	2.69 × 10^−8^	1.7702
	Thr-Ser	7.82 × 10^−17^	1.7545
	Ile-Hyp	2.43 × 10^−13^	1.6573
	Hyp-Arg	8.62 × 10^−8^	1.5598
	Arg-Ile	644 × 10^−6^	1.5183
	Val-Pro-Gln	1.29 × 10^−14^	1.5142
Other alkaloids and their derivatives	Amabiline	7.81 × 10^−16^	2.1625
Oligopeptide	Mucronine D	1.56 × 10^−11^	2.086
Other types of steroids and their derivatives	Cimiracemoside d	9.55 × 10^−19^	2.0417
	Taurocholic acid	7.89 × 10^−12^	1.5708
Dipeptide	Val-Gly	7.19 × 10^−10^	1.8839
	Lys-Pro	8.72 × 10^−8^	1.6304
	Lys-Ile	8.99 × 10^−7^	1.5577
Glycosides	Oleandomycin	2.03 × 10^−10^	1.8717
Organic acids and their derivatives	Candoxatrilat	2 × 10^−16^	1.8502
Macrolides and their derivatives	Ixabepilone	1.46 × 10^−8^	1.806
Fatty acyls	(S)-(-)-2-Hydroxyisocaproic acid	1.01 × 10^−8^	1.7731
	Leukotriene E4	1.41 × 10^−10^	1.7348
	1-Hexanol arabinosylglucoside	6.75 × 10^−12^	1.6402
Alpha amino acids and their derivatives	Cyclo(Pro-Leu)	3.8 × 10^−7^	1.6134
Nucleotides and their derivatives	Zalcitabine	5.4 × 10^−6^	1.6005
Coumarins and their derivatives	Dihydrocoumarin	7.63 × 10^−8^	1.5597

## Data Availability

The original contributions presented in this study are included in the article/Supplementary Materials. Further inquiries can be directed to the corresponding author.

## Supplementary Materials

The following supporting information can be downloaded at: https://www.mdpi.com/article/10.3390/foods14213733/s1, Table S1. Uniform design factors. Table S2. Volatile compound identified by GC-IMS. Table S3. Differentially expressed metabolites after fermentation. Figure S1. Effects of different addition levels of L. fermentum on pomegranate polyphenol content (a), total acid content (b), and soluble solids content (c). Figure S2. Effects of different addition levels of *L. casei* on pomegranate polyphenol content (A), total acid content (B), and soluble solids content (C). Figure S3. Effects of different addition levels of *L. rhamnosus* on pomegranate polyphenol content (A), total acid content (B), and soluble solids (C). Figure S4. Effects of different addition levels of *L. acidophilus* on pomegranate polyphenol content (A), total acidity (B), and soluble solids (C). Figure S5. Effects of different addition levels of *L. plantarum* on pomegranate polyphenol content (A), total acid content (B), and soluble solids (C). Figure S6. Polyphenol content (A), total acid content (B), and soluble solids content (C) after 12-h fermentation by different mixed-strain ratios (Factors 1–8, specific ratios shown in Table S1) and three single lactic acid bacteria. Different lowercase letters at the same time indicate significant differences (*p* < 0.05).
