# Supplementary material for: Development of a Mixed-Strain Fermentation Process for Sour Pomegranate: An Analysis of Metabolites and Flavor Compounds
Source: Foods. 2025 Oct 30;14(21):3733. doi: 10.3390/foods14213733 (PMC12607770; doi:10.3390/foods14213733)
Supplement: Supplementary file 1 [file foods-14-03733-s001.zip › foods-3909448-supplementary.pdf]

Supplementary material

Table S1. Uniform design factors

Factor level	*L. fermentum* X1/%	*L. plantarum* X2/%	*L. acidophilus* X3/%
1	42	34	26
2	4	36	29
3	45	35	33
4	47	33	29
5	43	38	30
6	46	37	31
7	41	32	27
8	45	31	28

Note: The data in Groups 1-8 in the table correspond to an orthogonal experimental design with three factors and eight levels, constructed using Minitab software.

Table S2. Volatile compound identified by GC-IMS

Count	Compound	CAS#	Formula	MW	RI	Rt [sec]	Dt [a.u.]
1	gamma -Butyrolactone	96-48-0	$C_4H_6O_2$	86.1	1643.9	1310.172	1.10077
2	Benzaldehyde	100-52-7	C_7H_6O	106.1	1509.1	967.338	1.15428
3	Acetic acid-M	64-19-7	$C_2H_4O_2$	60.1	1473.7	896.642	1.05368
4	Acetic acid-D	64-19-7	$C_2H_4O_2$	60.1	1473.7	896.642	1.15
5	1-nonanal-M	124-19-6	C_9H_1O	142.2	1395.1	762.276	1.47276
6	1-nonanal-D	124-19-6	C_9H_1O	142.2	1394	760.513	1.94137
7	1 -hexanol	111-27-3	C_6H_1O	102.2	1363.9	711.653	1.32762
8	2-ethylpyridine	100-71-0	C_7H_9N	107.2	1327.1	656.213	1.09386
9	Cyclohexanone	108-94-1	C_6H_1O	98.1	1334.7	667.185	1.14783
10	Benzene, butyl-	104-51-8	C_1H_1	134.2	1334.3	666.682	1.21562
11	2-Butanone, 3-hydroxy	513-86-0	$C_4H_8O_2$	88.1	1288.4	599.521	1.32984
12	1-octanal	124-13-0	C_8H_1O	128.2	1290.7	603.799	1.39871
13	1-Butanol, 3-methyl-M	123-51-3	C_5H_1O	88.1	1203.4	459.637	1.2421
14	2-butylfuran	4466-24-4	C_8H_1O	124.2	1140.6	370.046	1.18135
15	unidentified	unidentified	unidentified	0	1113.6	335.465	1.08816
16	1-Propanol, 2-methyl-M	78-83-1	C_4H_1O	74.1	1093.7	312.003	1.17314
17	1-Propanol, 2-methyl-D	78-83-1	C_4H_1O	74.1	1092.8	311.069	1.36348
18	Pyrrolidine	123-75-1	C_4H_9N	71.1	1042.8	265.275	1.04754
19	Propyl propanoate-M	106-36-5	$C_6H_1O_2$	116.2	1036.5	260.086	1.21719
20	unidentified	unidentified	unidentified	0	1024	250.136	1.13009
21	acrylonitrile-M	107-13-1	C_3H_3N	53.1	1023.8	249.972	1.04276
22	acrylonitrile-D	107-13-1	C_3H_3N	53.1	1012.3	241.157	1.08991
23	2,3-Butanedione	431-03-8	$C_4H_6O_2$	86.1	1012.3	241.157	1.18071
24	ethanol-M	64-17-5	C_2H_6O	46.1	970.9	212.674	1.04301
25	Ethanol-D	64-17-5	C_2H_6O	46.1	930	188.925	1.12745

Count	Compound	CAS#	Formula	MW	RI	Rt [sec]	Dt [a.u.]
26	unidentified	unidentified	unidentified	0	980.8	218.903	1.20838
27	Acetic acid ethyl ester-D	141-78-6	$C_4H_8O_2$	88.1	891.7	169.069	1.34032
28	unidentified	unidentified	unidentified	0	898	172.213	1.03053
29	Acetic acid ethyl ester-M	141-78-6	$C_4H_8O_2$	88.1	883.3	165.005	1.09667
30	Butanal	123-72-8	C_4H_8O	72.1	896.4	171.375	1.10859
31	2-propanone	67-64-1	C_3H_6O	58.1	830.7	141.703	1.1151
32	triethylamine	121-44-8	C_6H_1N	101.2	776	120.916	1.09016
33	2- butanol-M	78-92-2	C_4H_1O	74.1	1032.1	256.542	1.14756
34	2-pentyl furan	3777-69-3	C_9H_1O	138.2	1251.2	533.672	1.25162
35	2-Butanol-D	78-92-2	C_4H_1O	74.1	1040	262.976	1.33208
36	unidentified	unidentified	unidentified	0	1037.2	260.669	1.4525
37	Propyl propanoate-D	106-36-5	$C_6H_1O_2$	116.2	1034.8	258.744	1.58725
38	1-Butanol, 3-methyl-D	123-51-3	C_5H_1O	88.1	1202.5	458.376	1.48894

Table S3. Differentially expressed metabolites after fermentation.

Metabolite	VIP_pred_OPLS-DA	VIP_PLS-DA	FC (Post-ferm/Pre-ferm)	M/Z	ID	Class I	Class II
Presqualene diphosphate	4.4495	4.4513	2.6877	604.353	pos_3843	Terpenoids	Triterpenoids
Nummularine A	4.4998	4.4974	2.6017	648.380	pos_4044	Amino acids and derivatives	Cyclic peptides
Arg-Val-Phe	4.3325	4.3292	2.402	421.259	pos_4579	Others	Others
Met-Lys-Lys	4.0089	4.0103	2.2977	423.275	pos_10972	Others	Others
Cimicifugoside	4.173	4.1682	2.2008	692.406	pos_4190	Steroids and steroid derivatives	Other steroids and steroid derivatives
Amabiline	4.1035	4.1021	2.1625	338.193	pos_4173	Alkaloids and derivatives	Other alkaloids and derivatives
Mucronine D	4.1285	4.1269	2.086	694.421	pos_5158	Amino acids and derivatives	Oligopeptides
Cimiracemoside d	3.8261	3.8238	2.0417	696.437	pos_11306	Steroids and steroid	Other steroids and steroid derivatives

						derivatives	
Val-Gly	3.2327	3.2291	1.8839	175.108	pos_13925	Amino acids and derivatives	Dipeptides
Oleandomycin	3.7318	3.7321	1.8717	652.411	pos_4246	Carbohydrates and derivatives	Glycosides
Thr-Pro	3.4571	3.4613	1.8648	199.108	pos_14536	Others	Others
Candoxatrilat	3.7948	3.7927	1.8502	382.219	pos_11235	Organic acids and derivatives	Organic acids and derivatives
Ixabepilone	3.5772	3.5754	1.806	539.319	pos_12154	Others	Macrolides and derivatives
(S)-(-)-2-Hydroxyiso caproic acid	3.5831	3.5842	1.7731	131.070	neg_2078	Lipids	Fatty Acyls
Glu-Leu-Ser	3.2228	3.2235	1.7702	328.151	neg_7625	Others	Others
Thr-Ser	3.4218	3.4199	1.7545	189.087	pos_1624	Others	Others
Leukotriene E4	3.3951	3.3926	1.7348	472.275	pos_12477	Lipids	Fatty Acyls
Ile-Hyp	3.459	3.4577	1.6573	227.139	pos_2774	Others	Others
1-Hexanol arabinosylglucoside	3.329	3.3267	1.6402	414.234	pos_13906	Lipids	Fatty Acyls
Lys-Pro	3.2419	3.2436	1.6304	226.155	pos_14749	Amino acids and derivatives	Dipeptides
Lucidenic acid H	3.4578	3.455	1.6145	509.311	pos_4946	Terpenoids	Triterpenoids
Cyclo(Pro-Leu)	3.1152	3.1181	1.6134	211.144	pos_11949	Amino acids and derivatives	Alpha amino acids and derivatives
Zalcitabine	3.3661	3.3693	1.6005	212.103	pos_14659	Nucleotides and derivatives	Nucleotides and derivatives
Taurocholic acid	3.2848	3.284	1.5708	516.301	pos_12246	Steroids and steroid	Other steroids and steroid derivatives

						derivatives	
Hyp-Arg	3.0515	3.0449	1.5598	270.156	pos_785	Others	Others
Dihydrocoumarin	3.07	3.0657	1.5597	147.044	neg_2405	Coumarins and derivatives	Coumarins and derivatives
Lys-Ile	2.9247	2.9259	1.5577	242.186	pos_1750	Amino acids and derivatives	Dipeptides
Arg-Ile	2.8109	2.8139	1.5183	270.192	pos_1907	Others	Others
Val-Pro-Gln	3.1877	3.1868	1.5142	343.197	pos_14191	Others	Others
Ile-Glu	2.7158	2.7194	1.4754	241.119	neg_7143	Others	Others
9,10,18-Trihydroxyoctadecanoic acid	2.7226	2.7259	1.4717	331.249	neg_5092	Lipids	Fatty Acyls
Trans-EKODE-(E)-Ib	2.8025	2.8016	1.4483	311.221	pos_6299	Lipids	Fatty Acyls
6-Hydroxyhexanoic acid	2.7975	2.7962	1.4446	133.086	pos_11406	Organic acids and derivatives	Organic acids and derivatives
Glu-Phe	2.5842	2.5875	1.4401	275.104	neg_2317	Others	Others
Lys-His-Leu	2.8861	2.8838	1.4347	379.249	pos_4528	Others	Others
L-(-)-3-Phenyllactic acid	2.9884	2.9852	1.4344	165.055	neg_2406	Phenolic acids and derivatives	Phenylpropanoic acids and derivatives
Pro-Arg	3.01	3.0118	1.4298	254.161	pos_14575	Others	Others
Pro-Phe	2.7747	2.7749	1.4226	245.128	pos_4195	Others	Others
Chebi:69439	2.5208	2.5231	1.3487	197.128	pos_2504	Others	Others
16-Hydroxy-10-oxohexadecanoic acid	2.386	2.3853	1.3328	287.221	pos_6109	Lipids	Fatty Acyls
Ser-Pro-Lys	2.4902	2.488	1.3268	331.197	pos_1785	Others	Others
Tyr-Pro	2.6498	2.6487	1.3209	261.123	pos_12404	Others	Others

Roughanic acid	2.4828	2.4817	1.3199	251.200	pos_9593	Lipids	Fatty Acyls
Vernolic acid	2.3203	2.3229	1.3095	297.242	pos_9590	Lipids	Fatty Acyls
4-[3-(4,8-Dimethyl-3,7-nonadienyl)-5-3-methyloxiranyl]-2-butanone	2.2937	2.2964	1.3035	279.232	pos_6108	Terpenoids	Monoterpenoids
Indole-3-lactic acid	2.3457	2.3437	1.2943	204.066	neg_6344	Indoles and derivatives	Indoles and derivatives
Ile-Pro-Gln	2.3495	2.3493	1.2783	357.213	pos_13574	Others	Others
Pro-Ser	2.3795	2.378	1.2722	185.092	pos_14828	Others	Others
3-(4-Hydroxyphenyl) lactate	2.369	2.3668	1.2577	181.050	neg_1066	Phenolic acids and derivatives	Phenylpropanoic acids and derivatives
Netilmicin	2.4337	2.4332	1.2563	476.306	pos_12158	Carbohydrates and derivatives	Glycosides
Etherolenic acid	2.126	2.1247	1.2557	293.211	pos_9396	Lipids	Fatty Acyls
Asp-Leu	2.1064	2.1069	1.2237	227.103	neg_7877	Others	Others
Aleuretic acid	2.0338	2.0323	1.2209	287.221	pos_9885	Lipids	Fatty Acyls
O-glutarylcarnitine	2.3525	2.3536	1.1982	276.144	pos_14719	Lipids	Fatty Acyls
Pro-Ala	2.009	2.0083	1.197	187.108	pos_14199	Others	Others
15-Methoxyisodauc-3-16-ene-1beta,5alpha-diol	1.8701	1.8693	1.1831	269.211	pos_9879	Terpenoids	Sesquiterpenoids
Sclareolide	1.7885	1.7871	1.1748	251.200	pos_5823	Others	Others
Cyclo(His-Pro)	1.9406	1.9415	1.1702	235.119	pos_818	Others	Others

Methylmalonic acid	1.9892	1.9882	1.1657	117.018	neg_339	Organic acids and derivatives	Organic acids and derivatives
Val-Gly-His	2.1186	2.1196	1.1614	294.154	pos_983	Others	Others
3'-Hydroxygenistein	1.738	1.7358	1.1344	285.040	neg_5363	Flavonoids	Isoflavones
Carbidopa	1.6983	1.6978	1.1329	227.102	pos_1470	Phenolic acids and derivatives	Phenylpropanoic acids and derivatives
Gamma-delta-Dioxovaleric acid	1.573	1.5714	1.1269	151.003	neg_6778	Organic acids and derivatives	Organic acids and derivatives
Gln-Ile-Lys	1.6538	1.6533	1.1163	388.254	pos_12666	Amino acids and derivatives	Oligopeptides
1,6,8-trihydroxy-1H,3H-cyclohepta[c]furan-5-one	1.5068	1.505	1.1115	195.029	neg_2559	Others	Others
3-Hydroxy-1-indanone	1.4954	1.4928	1.1084	147.044	neg_8441	Others	Others
Trigonelline	1.2661	1.2775	1.1051	160.037	pos_15314	Alkaloids and derivatives	Other alkaloids and derivatives
Bergenin	1.504	1.5035	1.1004	349.057	neg_2165	Phenolic acids and derivatives	Benzoic acids and derivatives
Piperonylic acid	1.5718	1.5697	1.0988	165.018	neg_3317	Others	Others
Cedrin	1.512	1.5105	1.094	333.062	neg_2783	Flavonoids	Flavanol
Phthalic acid	1.4446	1.4421	1.0927	165.018	neg_3613	Phenolic acids and derivatives	Benzoic acids and derivatives
Normetanephrine	1.4776	1.475	1.0863	164.071	neg_535	Others	Others
6-Methyl	1.2534	1.2645	1.0858	377.069	pos_586	Carbohydrates	Other carbohydrates and

2-galloylgalactarate						and derivatives	derivatives
Quercetin	1.5708	1.569	1.081	301.035	neg_3319	Flavonoids	Flavonols
Luteolin	1.4202	1.4186	1.0801	285.041	neg_6708	Flavonoids	Flavones
9,10-Dihydro-4,5,7-trihydroxy-9,10-dioxo-2-anthroic acid	1.4135	1.4117	1.0766	299.020	neg_5937	Organic acids and derivatives	Organic acids and derivatives
4-(2-Nitroethyl)phenyl primeveroside	1.3484	1.3475	1.0726	484.145	pos_12062	Carbohydrates and derivatives	Glycosides
3,4-dihydroxymandelic acid	1.2864	1.2845	1.0709	165.018	neg_1873	Others	Others
Patulin	1.4072	1.4061	1.0688	153.018	neg_6777	Others	Others
Dihydrorobinetin	1.3215	1.3196	1.0683	303.051	neg_6817	Flavonoids	Flavanol
2-Methoxy-estradiol-17b 3-glucuronide	1.3399	1.3392	1.0661	511.251	pos_14244	Steroids and steroid derivatives	Other steroids and steroid derivatives
13(S)-HOTrE	1.2425	1.2423	1.0657	293.212	neg_4801	Lipids	Fatty Acyls
6-Hydroxykaempferol	1.0773	1.0777	1.0653	301.034	neg_2431	Flavonoids	Flavonols
2'-Hydroxygenistein	1.1377	1.1349	1.0611	285.040	neg_3680	Flavonoids	Isoflavones
Isodemethylwedelolactone	1.1838	1.18	1.061	299.020	neg_3425	Flavonoids	Isoflavones
Terephthalic acid	1.183	1.1814	1.0608	165.018	neg_6709	Phenolic acids and derivatives	Benzoic acids and derivatives
Erythromycin	1.292	1.2913	1.0583	738.447	pos_5282	Carbohydrates and derivatives	Glycosides

Gentisic acid	1.1254	1.1249	1.0572	137.023	pos_3088	Phenolic acids and derivatives	Benzoic acids and derivatives
3,5-dihydroxy-4-methoxybenzoic acid	1.1767	1.1764	1.0565	167.034	pos_12610	Phenolic acids and derivatives	Benzoic acids and derivatives
6-Hydroxyluteolin	1.2713	1.2697	1.0551	301.035	neg_7103	Flavonoids	Flavones
Tyramine	1.2922	1.2905	1.055	120.081	pos_14414	Others	Amines and derivatives
(-)-Epigallocatechin 7-glucuronide	1.2674	1.2671	1.0548	500.140	pos_3119	Flavonoids	Flavonoid glycosides
(Z) -N-Coumaroyl-5-(AA) hydroxyanthranilic acid	1.3088	1.3086	1.0547	643.152	neg_1569	Phenolic acids and derivatives	Cinnamic acids and derivatives
Diethyl dicarbonate	1.1274	1.1263	1.0535	183.029	neg_7528	Organic acids and derivatives	Organic acids and derivatives
Bellidifolin	1.2404	1.2391	1.0522	319.046	neg_1878	Others	Benzopyrans
4-(beta-D-glucosyloxy)-3-5-hydroxy-benzoic acid	1.2371	1.2362	1.0507	315.072	neg_1211	Carbohydrates and derivatives	Glycosides
2-Methoxy-1,4-benzoquinone	1.0167	1.0146	1.0506	183.029	neg_6399	Quinones	Benzoquinones
L-Phenylalanine	1.2139	1.2124	1.0491	166.086	pos_1292	Amino acids and derivatives	Phenylalanine and derivatives
Mgdg(8:0/8:0)	1.1637	1.163	1.0489	507.314	pos_5478	Others	Others
13-OxoODE	1.1312	1.1312	1.0461	295.226	pos_6739	Lipids	Fatty Acyls
Sativic acid	1.0923	1.0914	1.0458	329.233	neg_5046	Lipids	Fatty Acyls

Isovanillic acid	1.056	1.0554	1.0454	167.034	neg_6605	Phenolic acids and derivatives	Benzoic acids and derivatives
3'-(2"-Galloylglucosyl)-phloroacetophenone	1.1455	1.1442	1.0451	481.099	neg_7094	Carbohydrates and derivatives	Glycosides
3,3',4',5,5',8-Hexahydroxyflavone	1.0664	1.0654	1.044	317.030	neg_7667	Flavonoids	Flavonols
Stearidonic acid	1.0949	1.095	1.0422	277.216	pos_6738	Lipids	Fatty Acyls
9-Oxo-10,12-octadecadienoic acid	1.0152	1.015	1.0376	295.227	pos_9501	Lipids	Fatty Acyls
Protocatechuic acid	1	0.9984	1.0357	153.018	neg_700	Phenolic acids and derivatives	Benzoic acids and derivatives
Isomangiferin	1.0202	1.0203	1.035	481.099	neg_1565	Others	Benzopyrans
Genistin	1.0769	1.076	0.9683	433.113	pos_2649	Flavonoids	Isoflavones
6,7,4'-trihydroxyisoflavone	1.0103	1.0096	0.9654	271.060	pos_13068	Flavonoids	Isoflavones
Kaempferol 3-o-arabinoside	1.1129	1.1117	0.9648	419.097	pos_2816	Flavonoids	Flavonoid glycosides
gamma-Glu-Met	1.0303	1.0291	0.9611	279.101	pos_1279	Amino acids and derivatives	Dipeptides
Pisumionoside	1.0654	1.0662	0.9529	449.204	neg_2532	Terpenoids	Other terpenoids
2-[4-Methyl-2-(2-methylpropoxy)phenyl]-3-2-[(2-methylpropoxy)methyl]-oxirane	1.0327	1.0317	0.9525	293.211	pos_9577	Others	Phenol ethers

Dihydroalbocycline	1.0744	1.0732	0.9497	311.221	pos_6123	Others	Macrolides and derivatives
2-Linoleoyl Glycerol	1.2193	1.2166	0.9366	355.284	pos_8925	Lipids	Fatty Acyls
Methyl 15-hydroxy-7-labden-17-oate	1.311	1.3098	0.9325	337.273	pos_7668	Terpenoids	Diterpenoids
Oleoylglycerone phosphate	1.4836	1.483	0.9224	433.236	neg_4342	Others	Others
Adenosine	1.5636	1.5616	0.9205	268.104	pos_908	Nucleotides and derivatives	Nucleotides and derivatives
Glu-Gly-His	1.3132	1.3111	0.8967	342.139	pos_7804	Others	Others
Montanol	1.6318	1.6305	0.8867	353.268	pos_6618	Terpenoids	Sesquiterpenoids
9(s)-hode	1.7497	1.746	0.8511	295.228	neg_4842	Lipids	Fatty Acyls
Sqdg(18:3/18:3)	2.3365	2.3268	0.7661	837.483	neg_4142	Others	Others
Smgdg(o-18:4/18:3)	2.3082	2.3032	0.703	837.483	neg_4666	Others	Others
Polypodine B	1.898	1.8934	0.6749	535.270	pos_7341	Steroids and steroid derivatives	Other steroids and steroid derivatives
L-(+)-Arginine	3.3439	3.341	0.6317	175.119	pos_15262	Amino acids and derivatives	Alpha amino acids and derivatives

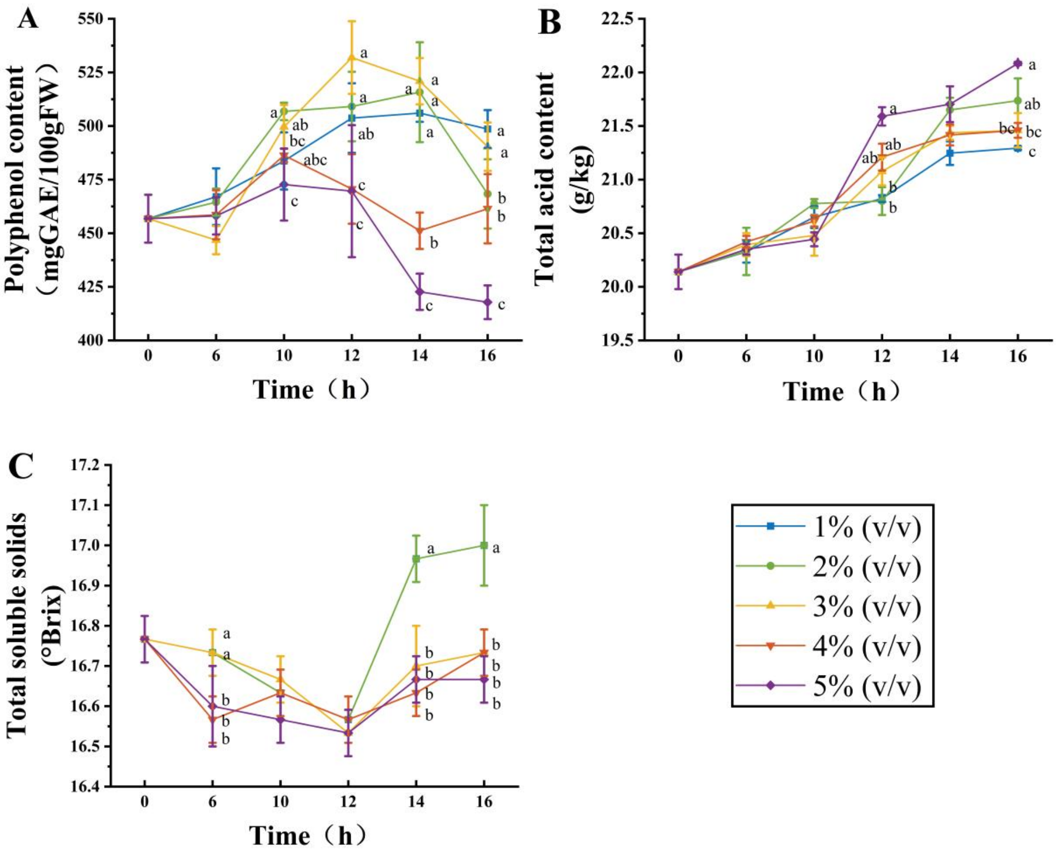

Fig. S1 Effects of different addition levels of *L. fermentum* on pomegranate polyphenol content (A), total acid content (B), and soluble solids content (C). Different lowercase letters at the same time indicate significant differences (P <0.05).

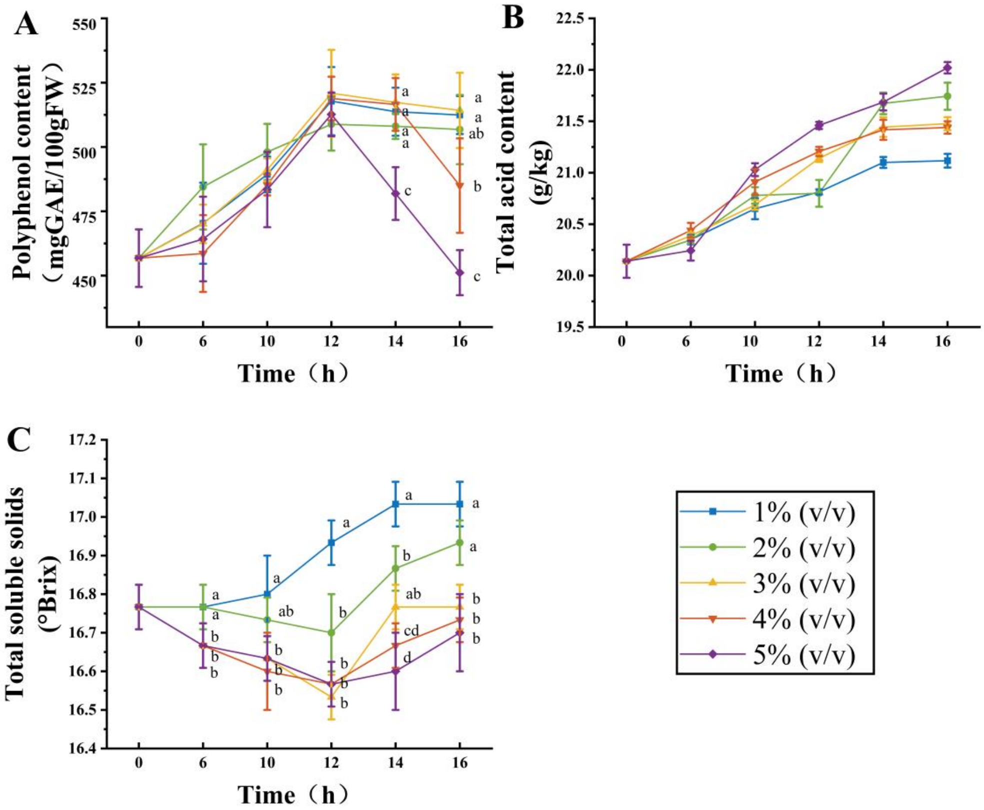

Fig. S2 Effects of different addition levels of *L.casei* on pomegranate polyphenol content (A), total acid content (B), and soluble solids content (C). Different lowercase letters for the same time point indicate significant differences (P <0.05).

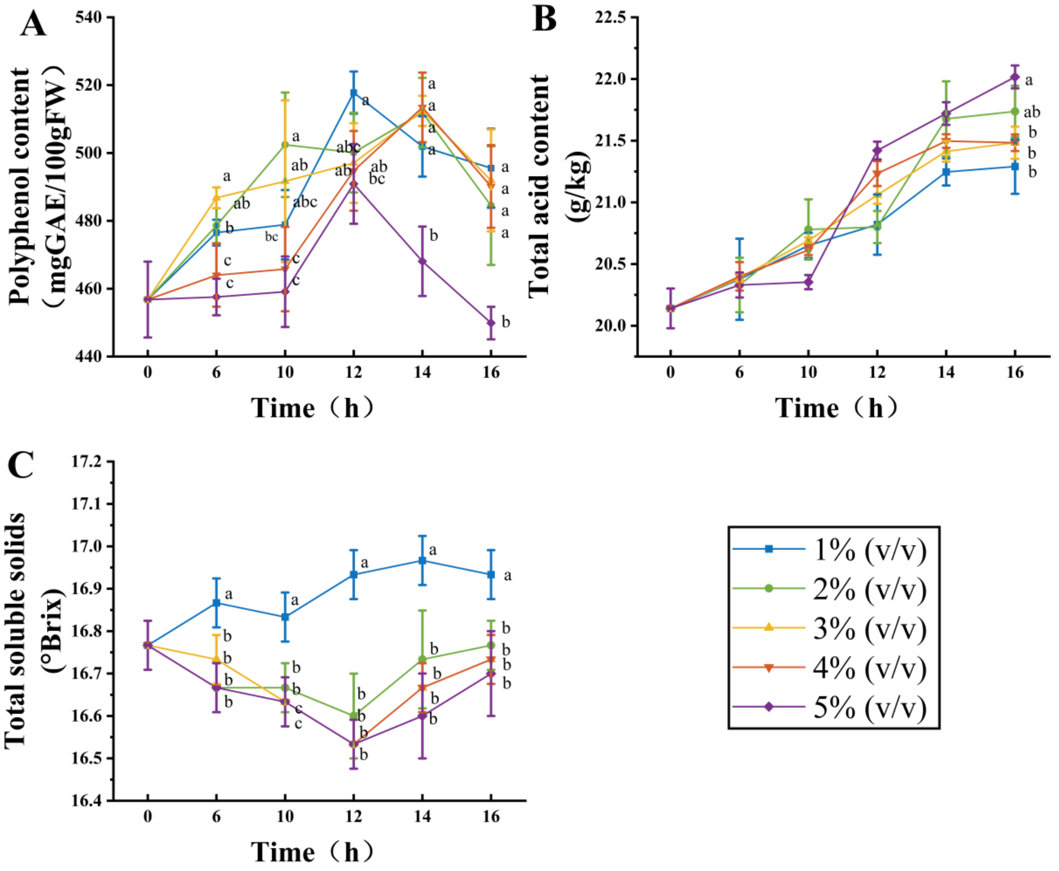

Fig. S3 Effects of different addition levels of *L. rhamnosus* on pomegranate polyphenol content (A), total acid content (B), and soluble solids (C). Different lowercase letters at the same time point indicate significant differences (p <0.05).

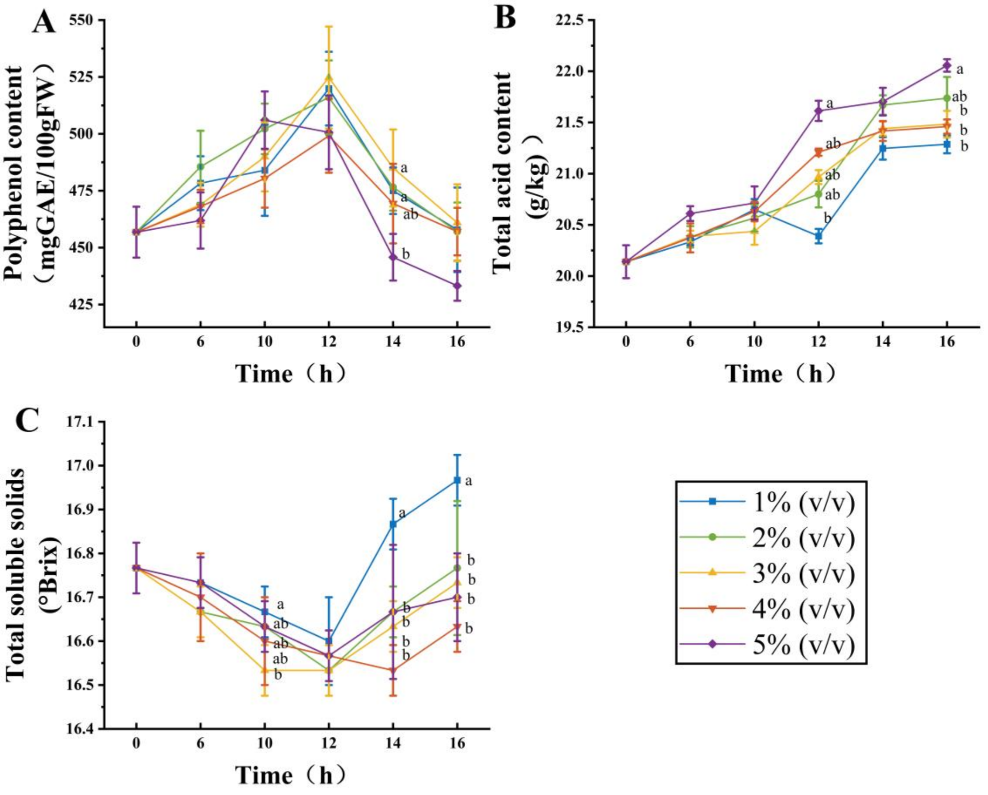

Fig. S4 Effects of different addition levels of *L. acidophilus* on pomegranate polyphenol content (A), total acidity (B), and soluble solids (C). Different lowercase letters at the same time point indicate significant differences (*P* <0.05).

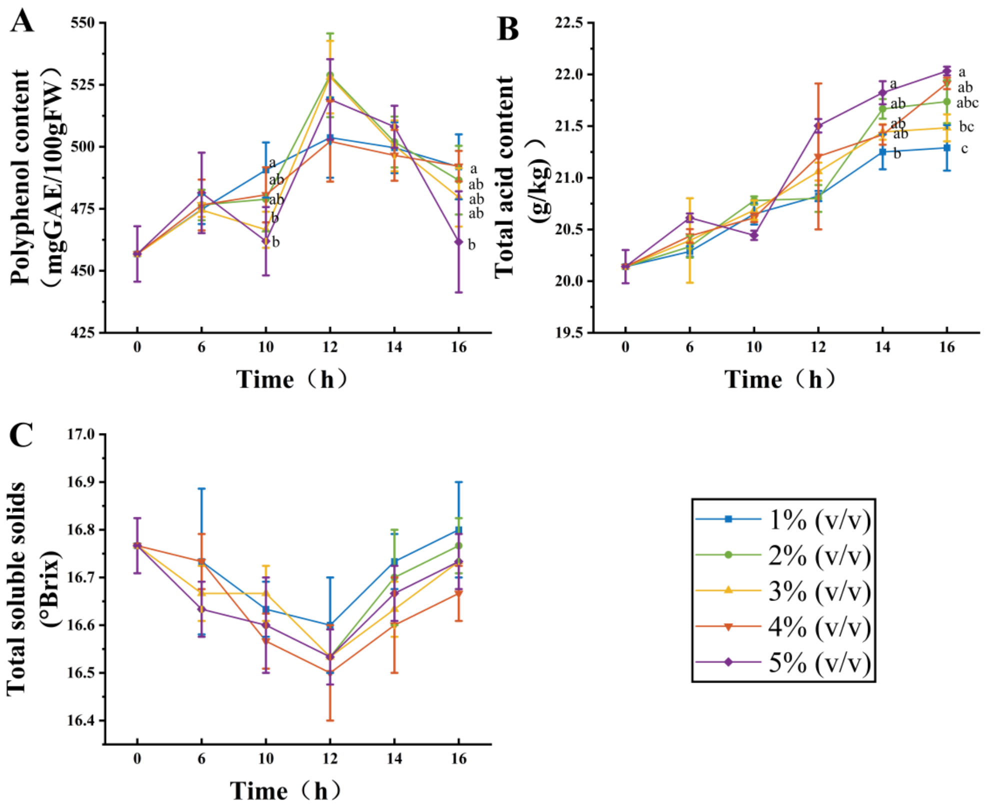

Fig. S5 Effects of different addition levels of *L. plantarum* on pomegranate polyphenol content (A), total acid content (B), and soluble solids (C). Different lowercase letters at the same time point indicate significant differences (*P* <0.05).

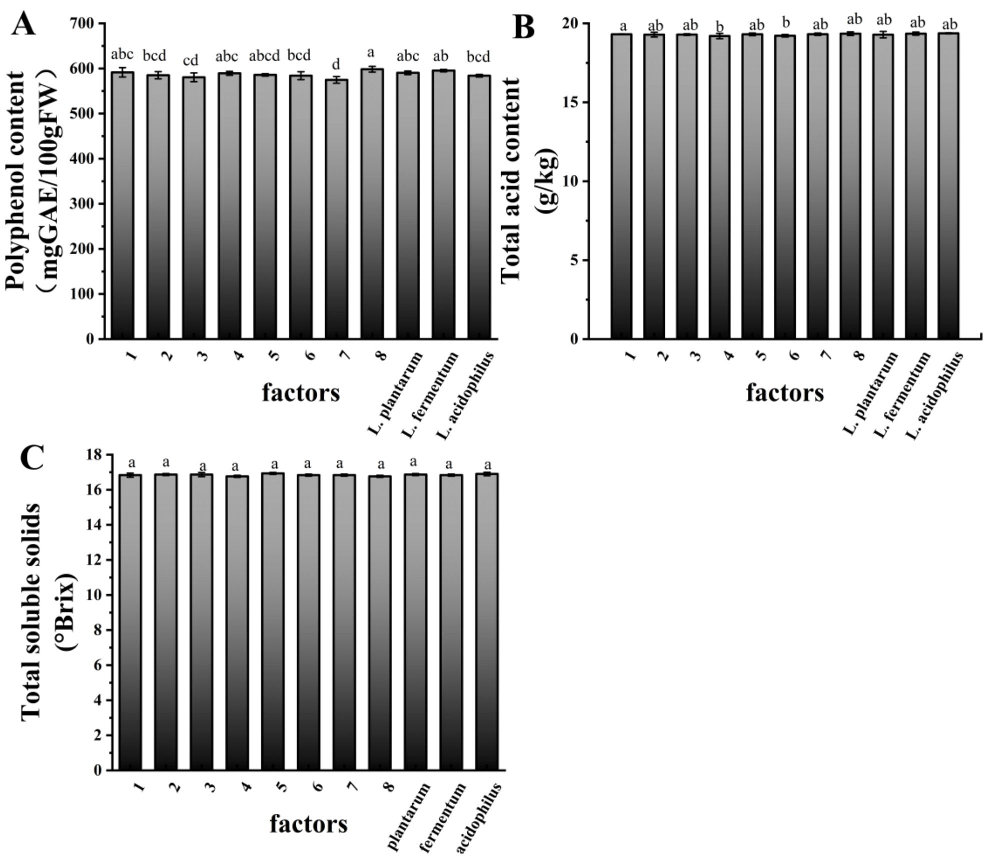

Fig. S6 Polyphenol content (A), total acid content (B), and soluble solids content (C) after 12-h fermentation by different mixed-strain ratios (Factors 1－8, specific ratios shown in Table S1) and three single lactic acid bacteria. Different lowercase letters at the same time indicate significant differences (*P* <0.05)
